# Predictive Factors of Successful Spinal Cord Stimulation in Patients with Chronic Pain: A Retrospective Cohort Study

**DOI:** 10.3390/brainsci15060614

**Published:** 2025-06-06

**Authors:** Yongjae Yoo, Hyungsang Roh, Jee Youn Moon, Eun Joo Choi, Francis Sahngun Nahm, Pyung Bok Lee

**Affiliations:** 1 Department of Anesthesiology and Pain Medicine, Seoul National University College of Medicine, Seoul 03080, Republic of Korea; rookie80@snu.ac.kr (Y.Y.); rowhs423@gmail.com (H.R.); jymoon0901@gmail.com (J.Y.M.); ejchoi@snubh.org (E.J.C.); hiitsme@hanmail.net (F.S.N.); 2 Department of Anesthesiology and Pain Medicine, Seoul National University Hospital, Seoul 03080, Republic of Korea; 3 Department of Anesthesiology and Pain Medicine, Seoul National University Bundang Hospital, Seongnam 13620, Republic of Korea

**Keywords:** spinal cord stimulation, chronic pain, complex regional pain syndrome, persistent spinal pain syndrome, neuropathic pain, pain management, quality of life, neurostimulation therapy

## Abstract

**Background:** Spinal cord stimulation (SCS) is applied for managing chronic intractable pain, but the factors predicting its effectiveness have not been extensively researched. Our study aimed to identify clinical variables that can predict the outcome of SCS. **Methods:** The electronic medical records of patients who received SCS for chronic intractable pain at two large tertiary teaching institutions in South Korea from 2008 to 2022 were reviewed. A successful outcome was characterized by attaining at least a 50% reduction in pain on the numerical rating scale (NRS) assessed at 6 months. Multivariable analysis was used to investigate the correlation between outcomes of SCS and clinical variables. **Results:** Of the 213 patients, 108 (50.7%) experienced successful outcomes at 6 months after SCS implantation. At 6 months, both the positive and negative outcome groups had significantly lower NRS pain scores than at baseline. Multivariable analysis revealed that male gender (*p* = 0.023) was an independent predictor of positive SCS outcomes; conversely, longer pain duration (*p* = 0.011) was a negative predictor. No significant adverse events associated with SCS were observed throughout the six-month follow-up duration. **Conclusions**: SCS could be an effective treatment for chronic intractable pain, including complex regional pain syndrome (CRPS) and persistent spinal pain syndrome (PSPS). More successful outcomes may be expected in male patients with a shorter duration of pain. Additional research is required to enhance patient selection processes and to identify clinical characteristics that contribute to improved long-term outcomes.

## 1. Introduction

Spinal cord stimulation (SCS) is a treatment for chronic pain, particularly effective for patients who are unresponsive to conventional methods. Introduced in the 1960s, SCS implants electrodes near the spinal cord to deliver electrical impulses. These impulses modulate pain signals before they reach the brain [1,2]. SCS creates an electric field between epidural metal contacts, altering the membrane’s electrical potential based on nearby tissue properties.

This technique is effective for treating conditions such as persistent spinal pain syndrome (PSPS, formerly known as failed back surgery syndrome) [3,4], complex regional pain syndrome (CRPS) [5], and other neuropathic pain [6,7].

Despite its growing clinical utility, there remain challenges and limitations associated with SCS. These include variability in patient response [8], complications related to device implantation [9], and the high cost of treatment [10]. Furthermore, not all patients achieve substantial or sustained pain relief with SCS [11], underscoring the importance of identifying factors that predict therapeutic efficacy.

The effectiveness of SCS is influenced by numerous patient-specific, technical, and psychosocial factors. For instance, the evidence suggests preimplantation characteristics such as baseline pain severity [12], duration of chronic pain [13], and psychological comorbidities, including depression and anxiety, may significantly impact treatment outcomes [14]. Additionally, proper patient selection through trial stimulation remains a cornerstone of the SCS implantation process, with the response during the trial phase often used as a proxy to predict long-term success [15]. However, variability in patient outcomes persists, even among those who demonstrate positive responses during the trial period.

Recent advances in SCS technology, like high-frequency and burst stimulation, allow for more personalized treatment options [16,17]. This evolution has also highlighted the need for a deeper understanding of predictive factors to optimize patient selection and improve outcomes. Factors such as pain etiology, lead placement accuracy, and patient engagement in multidisciplinary pain management programs are emerging as critical variables influencing SCS success [18,19].

Our objective was to identify predictors associated with the effectiveness of SCS in managing chronic pain. This study aimed to analyze the clinical variables associated with SCS to offer insights for enhancing patient outcomes and informing clinical decision-making. Identifying robust predictors will not only enhance the success rate of SCS but also ensure its cost effectiveness and broader applicability in chronic pain management.

## 2. Materials and Methods

### 2.1. Patients

This retrospective multicenter study was approved by the Institutional Review Board (IRB) of Seoul National University Hospital (SNUH IRB No. J-2308-127-1459) and Seoul National University Bundang Hospital (SNUBH IRB No. B-2307-840-101), Republic of Korea.

Following IRB approval, we reviewed the medical records of patients who had percutaneous SCS from 1 January 2008 to 31 December 2022. We enrolled patients with chronic intractable pain that did not respond to conservative treatment.

The SCS process began with a trial phase, where 1 (for unilateral pain) or 2 (for bilateral pain) percutaneous leads were placed in the cervical (for upper extremity pain) or thoracolumbar (for back and lower extremity pain) posterior epidural space under local anesthesia to test the therapy’s effectiveness. The entry location for the lead was determined using fluoroscopic images. After local anesthesia, the SCS lead was inserted into the epidural space and set to the optimal paresthesia coverage. We performed lateral fluoroscopy to allow the operator to document lead placement in the dorsal epidural space. All the SCS devices were implanted utilizing the conventional stimulation system. The frequency was calibrated within the range of 40–200 Hz; the amplitude was set between 0.5 and 3.0 V; and the pulse width was adjusted within the range of 60–350 μs.

Patients used an external stimulator for 5–14 days to assess pain relief, typically requiring at least 50% improvement to proceed. If successful, a permanent system was implanted with precise lead placement and a subcutaneous pulse generator connected to electrodes. The device was then programmed to optimize pain relief, and regular follow-ups were undertaken for proper functioning of the device and patient satisfaction.

The inclusion criteria were as follows: (1) patients with persistent pain despite conservative treatment; (2) age 19 years or older; (3) pain duration > 6 months.

The exclusion criteria were as follows: (1) not showing at least 50% improvement in pain reduction in the SCS trial process; (2) absence of follow-up data for 6 months.

### 2.2. Data Collection

We collected the demographic and clinical characteristics of the patients, including age; sex; body mass index (BMI) using height and weight; comorbid psychiatric conditions; litigation status; disease duration; diagnosis based on patient history; pain intensity measured by an 11-point numeric rating scale (NRS) pain score, with 0 indicating no pain and 10 indicating unbearable pain; smoking status; employment status; current medications; and the total prescription dose of opioids using average morphine equivalent daily dosage (MEDD, mg/day) at the baseline. Follow-up data, including NRS pain score, any changes in analgesics, and additional treatment for managing original pain, were also recorded.

SCS-related variables, including successful SCS trial, lead position, number of leads, possible procedure-related complications (e.g., lead migration, implantable pulse generator (IPG) failure, infection, hematoma, and pocket site pain), were also recorded.

The degree of patient satisfaction with the treatment was measured using a 5-point Likert scale (1 = very dissatisfied, 2 = somewhat dissatisfied, 3 = neither, 4 = somewhat satisfied, and 5 = very satisfied) at the 1-month follow-up visit after implantation; moreover, we calculated the proportion of “very satisfied” (score 5) and “somewhat satisfied” scores (score 4).

A positive (successful) outcome of the SCS procedure is defined as a reduction of 50% or more in the numeric rating scale (NRS) pain score compared with baseline, assessed at the 6-month follow-up post-implantation.

#### Statistical Analysis

All the statistical analyses were performed using IBM SPSS Statistics version 26.0. Continuous variables were summarized using the mean ± standard deviation (SD) or the median with interquartile range (IQR), based on the distribution determined by the Shapiro–Wilk test. Categorical variables were reported as frequencies and percentages.

Patients were grouped based on positive or negative outcomes using established criteria. For continuous variables, independent-sample t-tests or Mann–Whitney U tests were used as appropriate. For categorical variables, chi-square tests or Fisher’s exact tests were applied.

Subsequently, variables with a *p*-value < 0.20 in the univariate analysis, along with clinically relevant covariates, were entered into a binary logistic regression model to identify independent predictors of a successful outcome. The results were shown as odds ratios (ORs) with 95% confidence intervals (CIs). A *p*-value < 0.05 was deemed statistically significant. 

## 3. Results

**Figure 1:**
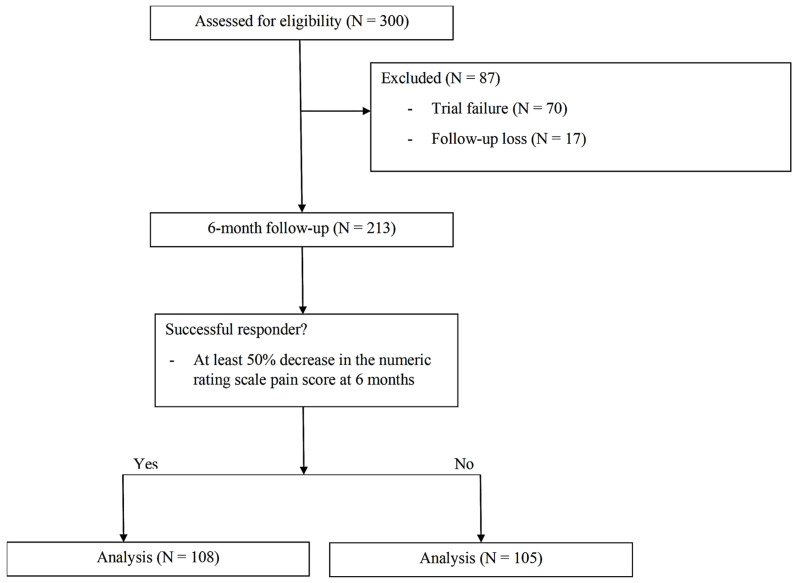
Consolidated standards of reporting trials (CONSORT) diagram.

We reviewed the medical records of 300 patients who underwent SCS trials from 1 January 2008 to 31 December 2022. A total of 87 patients were excluded from the study due to either the inability to proceed with implantation (N = 70) or lack of 6-month follow-up data (N = 17). Consequently, the analysis included 213 patients (Figure 1).

The patient demographics and characteristics are presented in Table 1. The most common diagnoses leading to SCS implantation were CRPS and PSPS, with CRPS type 1 accounting for more than 50% of the cases. Regarding the level of the SCS lead position, SCS leads were more commonly placed at the thoracolumbar level (74.2%) than at the cervical level (25.8%), indicating that a greater number of patients underwent SCS due to lower limb pain. Table 1 shows the demographic and clinical differences between groups with positive and negative outcomes based on predefined criteria. Of the 213 patients, 108 (50.7%) had positive outcomes by predefined criteria following SCS implantation. The gender ratio showed a significant difference between the successful outcome group and the negative outcome group.

The proportion of male patients was found to be higher in the positive outcome group than in the negative outcome group (63.0% vs. 50.5%; *p* = 0.048). In the positive outcome group, the proportions of “very satisfied” and “somewhat satisfied” were significantly higher (80.2% vs. 35.2%; *p* < 0.001). However, there were no significant differences in other variables between the two groups.

**Figure 2:**
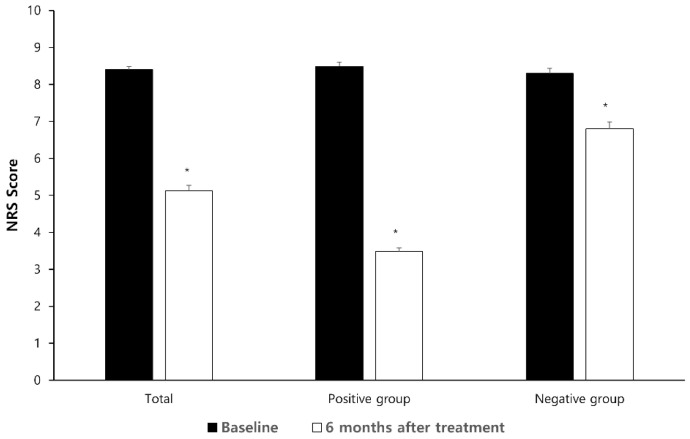
Changes in the NRS pain scores over time. The data are expressed as means ± standard deviations. * *p* < 0.001 when compared with baseline. NRS denotes the numerical rating pain scale.

Figure 2 shows the change in the patients’ NRS pain scores over time. The baseline NRS pain scores showed no significant difference between the two groups (8.49 ± 1.12 vs. 8.30 ± 1.35; *p* = 0.277). The overall NRS pain scores were significantly reduced after 6 months (5.12 ± 2.25; *p* < 0.001 compared with baseline). The NRS pain scores decreased at 6 months in both positive (3.48 ± 0.97; *p* < 0.001) and negative outcome groups (6.80 ± 1.92; *p* < 0.001).

Table 2 presents the clinical variables related to the outcomes based on univariable and multivariable regression analyses. These factors comprised male gender (*p* = 0.049), employment status (*p* = 0.112), and duration of pain (*p* = 0.010). Male gender (adjusted OR = 1.943; *p* = 0.023) independently predicted a successful outcome after SCS implantation, while longer pain duration (adjusted OR = 0.983; *p* = 0.011) predicted a negative outcome.

Regarding complications related to SCS implantation, lead migration (6.6% vs. 15.2%; *p* = 0.049) and surgical site pain (3.8% vs. 18.1%; *p* < 0.001) were significantly lower in the positive outcome group than in the negative outcome group. No significant device-related malfunctions occurred. Only mild infection was observed in 4 out of 213 patients (but there was no significant difference between the two groups (3 vs. 1, *p* = 0.621), and all the cases were well managed.

## 4. Discussion

While SCS offers an adjustable option for pain management, its success is contingent upon careful patient selection, thorough preoperative assessment, and ongoing postoperative follow-up. But the effectiveness of SCS may diminish over time, with a potential for complications like lead migration [18], infection [20], and the need for additional surgeries to adjust or replace the device [21]. Furthermore, previous studies investigating the duration of SCS effects have varied across patients and been influenced by factors such as the underlying pain condition, patient selection, and device programming [22,23]. However, research on clinical factors that affect the duration of the SCS effect is still lacking.

This study showed a significant reduction in pain intensity at 6 months following SCS implantation (*p* < 0.001 compared with baseline). A successful outcome, defined as a reduction of 50% or more in the NRS pain score from baseline at the 6-month follow-up, was reported in 50.7% of patients. This is somewhat lower than the overall result of about 67% from a comprehensive review that included 49 studies involving more than 2500 patients [24].

The trial-to-permanent conversion rate in SCS was 74.2% (213 out of 287 patients) in this cohort. Previous studies on SCS trials reported variable outcomes but generally suggested that approximately 60–80% of patients who undergo the trial phase of SCS proceed to receive a permanent implant [6,25,26]. The trial phase is designed to assess the effectiveness of the SCS system in alleviating pain, with the patient using a temporary lead and pulse generator for some periods. If the trial is successful and the patient experiences significant pain relief (usually defined as at least 50%), they are then considered for the permanent implant [27,28].

Nonetheless, for certain patients, the impact of SCS diminished completely over the follow-up period despite them having experienced significant pain relief during the initial trial [22,26]. A study evaluating both the short- and long-term effects of SCS found significant initial improvements in pain and health perceptions following the trial phase, but, over a seven-month follow-up, these benefits gradually diminished, resulting in outcomes like those of patients who did not proceed to permanent implantation [29]. Oakley et al. also found no correlation between the trial and permanent outcomes [30]. A recent study indicated that utilizing an SCS screening trial strategy provided no advantage in terms of pain severity or other outcomes compared with the approach of not conducting a trial [31]. These findings mean that consistent long-term pain relief is not guaranteed for all individuals with successful trials.

Clinical studies showed that the long-term effects of SCS vary depending on the indications [32]. While SCS provides initial pain relief, some studies have observed a decline in effectiveness over extended periods depending on the diagnosis. For example, a successful long-term outcome regarding pain relief in patients with PSPS reported that its effectiveness lasted for 72 months [33]. Another study involving patients with CRPS found that the effects were maintained for approximately 52 months [34]. These findings mean that the diagnosis that leads to SCS may be a factor affecting the effectiveness of SCS. However, in our study, the diagnosis did not significantly affect the duration of the effect for 6 months after SCS implantation.

The long-term success of SCS is sometimes measured by removal rates, with the lack of pain relief being the primary reason for device removal. Previous studies have not identified factors that predict SCS device explantation [8].

Several mechanisms may explain the reduced pain relief from SCS. First, neural desensitization could explain why the effectiveness of SCS is reduced. Over prolonged use, the nervous system may adapt to the continuous electrical stimulation, which may reduce the effectiveness of SCS. This phenomenon, known as neural desensitization, can result in decreased pain relief over time [35]. Second, changes in pain pathophysiology can occur over time. For instance, a transition from nociceptive to neuropathic pain can alter the response to SCS, leading to decreased efficacy [36]. Sometimes psychological factors such as depression and anxiety can affect the perception of pain and the effectiveness of pain management strategies, including SCS [37].

In our study, the group with positive outcomes demonstrated a greater reduction in pain intensity and higher levels of satisfaction than the group with negative outcomes. It was found that male gender (*p* = 0.023) was an independent predictor of positive SCS outcomes. Previous studies have reported similar findings on factors related to effective SCS [13,34].

For the clinical variables, we found that gender was associated with the effectiveness of SCS implantation. This could be due to several factors, both biological and psychosocial. Research on pain in patients with spinal cord injury (SCI) indicates that females experience a higher prevalence of nociceptive pain than males. This suggests that, despite similar descriptions of pain, men and women may perceive pain differently due to varying pathophysiologies, potentially leading to different baseline responses to SCS stimulation [38]. And male patients showed differences in employment status and worker’s compensation rates compared with females in our study. One possibility is that these results may be due to potential secondary benefit issues.

The duration of chronic pain before spinal cord stimulation (SCS) is a significant factor in determining its long-term effectiveness for patient selection and predicting outcomes. We found that longer pain duration (*p* = 0.011) was a negative predictor of successful SCS. Similar results were reported in a previous study examining patients with chronic pain who underwent SCS implantation, finding that those with a shorter duration of pain prior to the procedure experienced more significant and sustained pain relief than those with longer pain histories [39]. While individual responses can vary, initiating SCS treatment earlier in the course of chronic pain may enhance the likelihood of sustained pain relief. Therefore, clinicians should consider the duration of a patient’s pain when assessing the potential long-term benefits of SCS therapy.

This study has the following limitations: The retrospective design of this study presents a limitation. Additional treatments such as physical therapy, injections, and medication were not controlled, and these factors could confound pain outcomes. However, patients who underwent SCS implantation continued follow-up at the same hospital after the procedure and had their pain managed under the same conditions in this study. Another possible drawback is that it does not check other parameters, such as DN4 (Douleur Neuropathique en 4 Questions) for neuropathic pain screening. Another limitation of our study is the lack of a control group. Although we investigated procedure-related complications, this study did not measure the SCS-related side effects. The outcome of SCS treatment may be influenced by psychological factors, and SCS treatment itself may influence these factors. Further research is needed to elucidate the influence of psychological factors on SCS outcome. And we did not consider cost effectiveness when assessing the effectiveness of SCS. Furthermore, considering SCS use duration in other studies [34], 6 months of follow-up might be inadequate to evaluate long-term effectiveness. Therefore, a multicenter study with a large sample and long-term follow-up would be needed to clarify these causes. Nonetheless, our study may be more suitable for examining the effect in chronic intractable pain because SCS trials were undergone only by the patients who complained of severe pain with an NRS pain score of 7 or higher according to the National Health Insurance criteria for SCS trial coverage in South Korea.

## 5. Conclusions

Spinal cord stimulation (SCS) is a treatment option for individuals experiencing chronic intractable pain, such as complex regional pain syndrome (CRPS) and persistent spinal pain syndrome (PSPS). Our research indicates that male patients with a shorter duration of pain may experience more successful outcomes. Further research is required to optimize patient selection and identify clinical characteristics that contribute to better long-term results.

## Figures and Tables

**Table 1:** Demographics and baseline clinical characteristics: data are presented as number (%), means (±SD), or median (interquartile range). BMI, body mass index; NRS, numeric rating scale; CRPS, complex regional pain syndrome; PSPS, persistent spinal pain syndrome; MEDD, morphine equivalent daily dosage.

Variables	Total (N = 213)	Positive Outcome (N = 108)	Negative Outcome (N = 103)	*p* Value
Age (yrs)	53.00 ± 17.59	53.23 ± 17.79	52.76 ± 17.47	0.846
Sex (M/F)	120/93	68/40	52/53	0.048
BMI (Kg/m^2^)	24.99 ± 4.31	24.87 ± 3.97	25.11 ± 4.65	0.676
Comorbid psychiatric disorder	89 (41.8)	47 (43.5)	42 (40.0)	0.677
Litigation status	83 (38.0)	41 (38.0)	42 (40.0)	0.780
Disease duration (months)	25.0 (14.5–36.0)	25.0 (20.5–27.0)	26.0 (12.00–46.0)	0.174
Diagnosis				
CRPS type 1	114 (53.5)	56 (51.9)	58 (55.2)	0.681
CRPS type 2	27 (12.7)	16 (14.8)	11 (10.5)	0.412
PSPS	39 (18.3)	19 (17.6)	20 (19.0)	0.860
Others	33 (15.5)	17 (15.7)	16 (15.2)	>0.999
Smoking	46 (21.6)	22 (20.4)	24 (22.9)	0.740
Employment	47 (22.1)	19 (17.6)	28 (26.7)	0.137
Lead position				0.876
Cervical	55 (25.8)	27 (25.0)	28 (26.7)	
Thoracolumbar	158 (74.2)	81 (75.0)	77 (73.3)	
Number of leads				0.463
1	147 (69.0)	72 (66.7)	75 (71.4)	
2	66 (31.0)	36 (33.3)	30 (28.6)	
MEDD (mg/day) at baseline	38.80 ± 55.61	34.45 ± 54.66	43.28 ± 56.47	0.248
Preoperative NRS	8.00 (8.00–10.00)	8.00 (8.00–10.00)	8.00 (7.00–10.00)	0.384
Patient satisfaction	122 (57.8)	85 (80.2)	37 (35.2)	<0.001

**Table 2:** Clinical variables related to outcomes using regression analysis (r^2^ = 0.093). Backward elimination retained variables with *p*-value < 0.05. OR, odds ratio; CI, confidence interval; BMI, body mass index; CRPS, complex regional pain syndrome; PSPS, persistent spinal pain syndrome; NRS, numeric rating scale.

	Univariable Analysis	Multivariable Analysis		
	OR (95% CI)	*p*-Value	OR (95% CI)	*p*-Value
Age	1.002 (0.986, 1.017)	0.845	1.943 (1.096, 3.444)	0.023
Male	1.733 (1.003, 2.993)	0.049		
BMI	0.987 (0.926, 1.051)	0.675		
Litigation	0.918 (0.529, 1.592)	0.761		
Pain duration	0.984 (0.972, 0.996)	0.010	0.983 (0.971, 0.996)	0.011
Employment	0.587 (0.304, 1.133)	0.112	0.507 (0.254, 1.013)	0.054
Diagnosis				
CRPS type 1	0.873 (0.509, 1.496)	0.620		
CRPS type 2	1.486 (0.655, 3.373)	0.343		
PSPS	0.907 (0.453, 1.817)	0.784		
Others	1.039 (0.495, 2.184)	0.919		
Lead position				
Cervical	0.917 (0.496, 1.694)	0.781		
Thoracolumbar	1.091 (0.590, 2.015)	0.781		
Psychiatric comorbidity	1.156 (0.670, 1.993)	0.603		
Opioid use	0.698 (0.388, 1.256)	0.230		
Baseline NRS pain score (0–10)	1.129 (0.908, 1.405)	0.275		

## Data Availability

The Data are not publicly available due to the inclusion of sensitive patient information but are available from the corresponding author on reasonable request and after approval of the institutional review board.

## Abbreviations

SCS	Spinal cord stimulation
CRPS	Complex regional pain syndrome
PSPS	Persistent spinal pain syndrome
NRS	Numeric rating scale
MEDD	Morphine equivalent daily dosage
